# Curcumin analog WZ26 induces ROS and cell death via inhibition of STAT3 in cholangiocarcinoma

**DOI:** 10.1080/15384047.2022.2162807

**Published:** 2023-01-16

**Authors:** Minxiao Chen, Chenchen Qian, Bo Jin, Chenghong Hu, Lingxi Zhang, Minshan Wang, Bin Zhou, Wei Zuo, Lijiang Huang, Yi Wang

**Affiliations:** a Department of Gastroenterology, the Affiliated Xiangshan Hospital of Wenzhou Medical University, Ningbo, China; b Chemical Biology Research Center, School of Pharmaceutical Sciences, Wenzhou Medical University, Wenzhou, China; c Department of Pharmacy, the First Hospital of Xiangshan, Ningbo, China; d Department of Hepatopancreatobiliary Surgery, the Second Affiliated Hospital of Wenzhou Medical University, Wenzhou, China

**Keywords:** Curcumin analog, mitochondrial apoptosis, cholangiocarcinoma, ROS, STAT3

## Abstract

Cholangiocarcinoma (CCA) is an aggressive biliary epithelial tumor with limited therapeutic options and poor prognosis. Curcumin is a promising active natural compound with several anti-cancer properties, though its clinical uses remain hindered due to its poor bioavailability. We recently synthesized curcumin analogs with multifunctional pharmacological and bioactivities with enhanced bioavailability. Among these novel curcumin analogs, WZ26 is a representative molecule. However, the anti-tumor effect of WZ26 against CCA is unclear. In this study, we evaluated the anti-tumor effect of WZ26 in both CCA cells and CCA xenograft mouse model. The underlying molecular anti-cancer mechanism of WZ26 was also studied. Our results show that WZ26 significantly inhibited cell growth and induced mitochondrial apoptosis in CCA cell lines, leading to significant inhibition of tumor growth in xenograft tumor mouse model. Treatment of WZ26 increased reactive oxygen species (ROS) generation, subsequently decreased mitochondrial membrane potential and inhibited the phosphorylation of signal transducer and activator of transcription 3 (STAT3), thereby inducing G2/M cell cycle arrest and cell apoptosis. Pretreatment of N-acetyl cysteine (NAC), an antioxidant agent, could fully reverse the WZ26-induced ROS-mediated changes in CCA cells. Our findings provide experimental evidence that curcumin analog WZ26 could be a potential candidate against CCA via enhancing ROS induction and inhibition of STAT3 activation.

## Introduction

Cholangiocarcinoma (CCA) is one of the rare devastating cancers with limited therapeutic options and poor prognosis. It arises from varying regions of epithelial cells lining of biliary tract with markers of cholangiocyte differentiation.^1^ CCA is anatomically classified as extrahepatic, intrahepatic, perihilar, and distal CCA.^2^ The incidence of CCA has been increasing worldwide, relatively high in the Asian countries and parts of South America.^3^ Current management for CCA includes systemic chemotherapy, targeted radiation therapy, and surgical intervention with sub-optimal and modest outcomes.^4^

Elevated reactive oxygen species (ROS) are found in many cancer cells, including CCA.^5^ ROS generated as oxysterols (cholesterol oxidation products) from biliary cholesterol stimulates tumor development milieu in the biliary tract.^6^ Oxysterols also promote proliferation, migration, and invasion of CCA cells via Hedge-hog signaling.^7^ Also, ROS, which is derived from cancer cell metabolism, activates oncogenic signaling and promotes the transcription of transcription factors.^8^ Signal transducer and activator of transcription 3 (STAT3) is a principal transcription factor that plays important roles in the diverse cellular activities in CCA cancer cells.^9,10^ Overactivation of STAT3 in the carcinogenic microenvironment initiates the oncogenesis of CCA, further leading to the increase in the tumor cell survival and invasion, angiogenesis, and apoptosis resistance.^11–13^ However, high levels of ROS are also vulnerable to cancer cells, including arresting the cell cycle and activating apoptosis.^14^ Many chemotherapeutic agents exert their anti-tumor activities through the synthesis of ROS via mitochondrial pathway.^15,16^ Thus, increasing the levels of ROS in CCA cells could be a potential strategy for the treatment of CCA.

Natural products and their derivatives have the potential to prevent various cancers via inducing apoptosis and cell cycle arrest through pleiotropic mechanisms. Curcumin has been reported to have an extensive range of biological activities including anti-diabetes,^17^ anti-obesity,^18^ anti-oxidant,^19^ and anti-inflammatory.^20^ Curcumin possesses chemo-preventive or chemo-therapeutic activities against various cancers including CCA.^21^ Although curcumin possesses many chemo-therapeutic effects, the clinical uses of curcumin remain hindered due to its low potency and poor bioavailability.^22^ Recently, our lab synthesized curcumin analogs with multifunctional pharmacological and bioactivities with enhanced bioavailability.^23^ The purpose of this study was to examine the toxic effect of a curcumin analog, (1E,4E)-1-(3-bromo-4-hydroxyphenyl)-5-(4-hydroxy-3-methoxyphenyl)penta-1,4-dien-3-one (WZ26), on CCA cells *in vitro* and in CCA xenograft mouse model. Our results demonstrate that WZ26 increased ROS production via reducing mitochondrial membrane potential, and inhibited activation of STAT3 signaling, thereby inducing apoptosis and cell cycle arrest in CCA cancer cells. WZ26 could be a potential candidate for the treatment of CCA.

## Methods

### Chemicals and reagents

**Figure 1.**
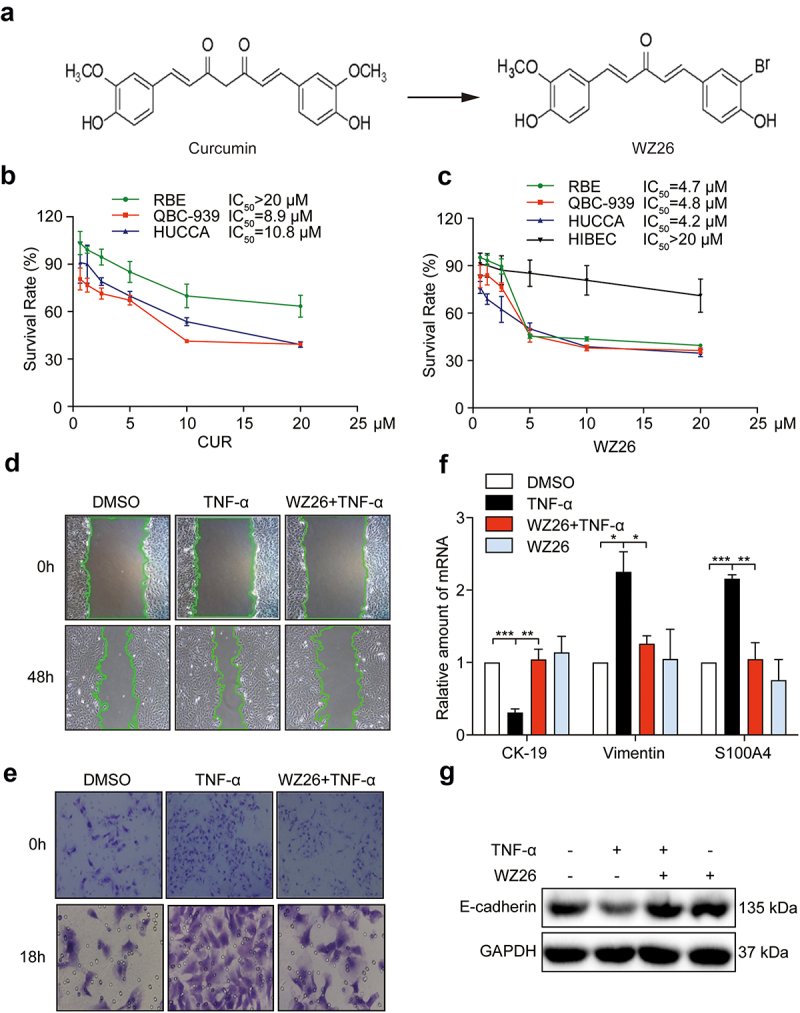
WZ26 inhibits cell growth, migration, and invasion in cholangiocarcinoma cells. (a) Chemical structure of curcumin (CUR) and WZ26. (b-c) RBE, QBC-939, and HUCCA cells were treated with various doses of curcumin (0.625, 1.25, 2.5, 5, 10, and 20 μM) and WZ26 (0.625, 1.25, 2.5, 5, 10, and 20 μM) or vehicle control (DMSO, 1 μL) for 24 h. HIBEC cells, as the normal cell control, were treated with WZ26 (0.625, 1.25, 2.5, 5, 10, and 20 μM) or vehicle control (DMSO, 1 μL) for 24 h. Cell viabilities were determined using MTT assay. IC_50_ values of CUR and WZ26 were obtained using GraphPad Prism 7.0. (d) The representative migration photos. QBC-939 cells were treated with WZ26 (2.5 μM) or vehicle control (DMSO, 1 μL) for 30 min, followed by treatment of TNF-α (10 ng/mL) for 48 h. (e) Representative images of QBC-939 cells stained with crystal violet (blue) that have migrated across Matrigel-coated trans-well filters. QBC-939 cells were treated as described in panel d. Cells were stimulated with TNF-α for 18 h for invasion analysis. (f) QBC-939 cells were treated as described in panel d. mRNA levels of epithelial–mesenchymal transition (EMT) markers such as CK-19, vimentin and S100A4 were evaluated using RT-qPCR. (g) QBC-939 cells were treated as described in panel d. Protein levels of E-cadherin was determined using Western blot analysis. GAPDH was used as loading control. All statistical data were shown as mean ± SEM, n = 3. *, *P* < .05; **, *P* < .01; ***, *P* < .001.

Annexin V-FITC/Propidium Iodide apoptosis and PI/RNase cell cycle detection kits were purchased from BD Biosciences (Franklin Lakes, NJ). TNF-α was obtained from PeproTech Inc. (Rocky Hill, NJ). 3-(4,5-Di-methylthiazol-2-yl)-2,5-diphenyl-2 tetrazolium bromide (MTT), ROS fluorescence probes DCFH-DA, mitochondrial integrity probe JC-1, MitoTracker Green, Lipid peroxidation MDA assay kit, and Caspase-3/9 activity assay kits were purchased from Beyotime Biotech (Nantong, China). Antibodies for E-cadherin, Cle-caspase 3, CDC2, CyclinB1, JC-1, Ki-67, Cytochrome c, GAPDH, and the secondary horseradish peroxidase-conjugated antibody were obtained from Santa Cruz Biotechnology (Santa Cruz, CA). Antibodies for pro-caspase 3, p-STAT3, and STAT3 were purchased from Cell Signaling Technology (Danvers, MA). N-acetyl cysteine (NAC), curcumin (CUR), and LPS were purchased from Sigma-Aldrich (St. Louis, MO, USA). WZ26 (>98% purity) was synthesized in our lab as described previously.^24^ The chemical structure of WZ26 and CUR is shown in Figure 1a. CUR and WZ26 were dissolved in DMSO and sodium carboxymethyl cellulose (CMC-Na, 1%) for *in vitro* and *in vivo* experiments, respectively.

### Cell lines and maintenance

Human cholangiocarcinoma cell line (RBE, Cat#: TCHu179) and macrophage cell line (RAW 264.7, Cat#: TCM13) were purchased from Cell Bank of Shanghai Institutes for Biological Sciences (Shanghai, China). Normal human biliary epithelial cell line (HIBEC) and human cholangiocarcinoma cell lines (QBC-939 and HUCCA) were obtained from School of Pharmaceutical Sciences, Wenzhou Medical University (Wenzhou, China). All cells were cultured in RPMI 1640 (Gibco, Eggenstein, Germany) supplemented with 10% heat-inactivated FBS (Hyclone, Logan, UT), 100 U/mL penicillin, and 100 mg/mL streptomycin (Mediatech Inc., Manassas, VA) in a humidified atmosphere of 5% CO_2_ at 37°C.

### MTT assay

Normal human biliary epithelial cells or cholangiocarcinoma cells were seeded at a density of 5 × 10^3^ cells per well into 96-well plates followed by 24-h cell adhesion. Cells were treated with CUR or WZ26 dissolved in DMSO and diluted with respective culture medium at different concentrations. After 24-h treatment, freshly prepared MTT reagent was added, and cells were incubated for 4 h. Formazan crystals resulting from mitochondrial enzymatic activity were dissolved with DMSO and absorbance was determined at 490 nm. Each experiment was done in triplicates and repeated three times. Results were expressed as a percentage of vehicle control. IC_50_ values of each compound were calculated using GraphPad Pro 5.0 (San Diego, CA).

### Scratch wound assay

QBC-939 cells were seeded at a density of 4 × 10^5^ cells/well into 6-well plates. When the confluence reached 90%, scratches were made on a single-cell surface with 200 µL pipette tips. Then, the cell medium was changed to fresh serum-free medium with mitomycin C (5 μg/mL). QBC-939 cells were treated with WZ26 (2.5 μM) or vehicle control (DMSO, 1 μL) for 30 min, followed by treatment of TNF-α (10 ng/ml) for 48 h. Images of cells were captured using a microscope (×100; Nikon, Tokyo, Japan) at 0 and 48 h after treatment.

### Matrigel invasion assay

Twenty-four-well transwell plates with 8 μM pore size (Corning Costar Corp., Shanghai, China) were coated with 10 μL Matrigel (diluted 1:9 with serum-free medium, BD Biosciences, Franklin Lakes, NJ) for each well. QBC-939 cells (2 × 10^5^) suspended in 200 μL serum-free RPMI 1640 culture medium were plated into the upper Matrigel-coated chamber. The bottoms chamber was loaded with 500 μL of RPMI 1640 medium containing 10% FBS and the cells were cultured at 37°C for 18 h. Invaded cells in the bottom chamber were fixed with 1% methanol for 15 min, followed by staining with crystal violet for 25 min, and washed thrice with PBS. The images were visualized under a light microscope (Nikon, Tokyo, Japan) and photographed.

### Cell apoptosis assay

Apoptosis was determined using Annexin V-FITC/PI apoptosis detection kit according to the manufacturer’s protocol. RBE, QBC-939, and HUCCA cells were seeded at a density of ×10^5^ cells/well into 6-well plates. After being treated with WZ26 (5, 10, or 15 μM), CUR (20 μM) or vehicle control (DMSO, 1 μL) for 24 h, cells were harvested, washed twice with ice-cold PBS, and evaluated for apoptosis by double staining with FITC conjugated Annexin V and propidium iodide (PI) in binding buffer for 30 min using a FACS Calibur flow cytometer.

### Cell cycle analysis

Cell cycle stages and nuclear DNA contents were determined using PI staining and flow cytometry assay. The cells were collected, fixed with 75% ice-cold ethanol and stored at −20°C for 24 h and washed with PBS, then stained with PI [50 μg/mL PI and 10 μg/mL ribonuclease (RNase) in PBS] at 4°C for 20 min in the dark place. The cells were washed and subjected to an FACS-Caliber flow cytometric analysis of the DNA content. Cell fractions in the G2/M phase were used for statistical analysis using the Flow Jo 7.6 software (TreeStar, San Carlos, CA).

### Western blotting assay

Lysates from cells or tumor tissues were prepared for protein expression using the Bradford assay (Bio-Rad, Hercules, CA). Proteins were separated by SDS-PAGE and blotted to PVDF transfer membranes. The blots were blocked for 2 h at room temperature using fresh 5% nonfat milk in TBST and incubated with specific primary antibodies overnight at 4°C. Followed by incubation with secondary antibodies for 1 h, the immunoreactive bands were visualized by using ECL kit (Bio-Rad). The density of the immunoreactive bands was analyzed using Image J computer software (National Institute of Health, MD).

### RNA isolation and real-time quantitative PCR

Total RNA was isolated from cells using TRIZOL (Invitrogen, Carlsbad, CA) according to the manufacturer’s protocol. Reverse transcription and quantitative PCR were performed using M-MLV platinum RT-qPCR kit (Invitrogen, Carlsbad, CA). Real-time qPCR was performed using the Eppendorf Real plex 4 (Eppendorf, Hamburg, Germany). Primers for genes including CK-19, vimentin, S100A4, and β-actin were synthesized in Invitrogen (Invitrogen, Shanghai, China). The relative amount of each gene was normalized to the amount of β-actin.

### Caspase-3 and caspase-9 activity assay

Caspase-3 and 9 activities in cell lysates were determined using Caspase-3 and 9 activity kits (Beyotime Biotech Nantong, China) according to the manufacturer’s protocol. Caspase-3 and 9 activities were normalized by the protein concentration of the corresponding cell lysate and expressed in enzymatic units per mg of protein.

### Determination of intracellular ROS

Intracellular ROS contents were measured by flow cytometry utilizing DCFH-DA. Cells were stained with 10 μM DCFH-DA at 37°C for 30 min in the dark place. DCF fluorescence (produced in the presence of ROS) was analyzed using flow cytometry and fluorescence microscope (Nikon, Tokyo, Japan).

### Determination of mitochondrial membrane potential (MMP) and cytochrome C release

MMP was determined using JC-1 staining according to the manufacturer’s protocol. The cell mitochondrial membrane potential (Δψm) was examined by fluorescence microscope using JC-1 (Beyotime Biotech, Nantong, China) as a specific probe. Images acquired from monomer and aggregate were merged and viewed under a Nikon fluorescence microscope. Evaluation of the subcellular localization of cytochrome c was done by using fluorescence imaging of cells double-labeled with MitoTracker Green and cytochrome c antibody. After treatment, cells were incubated with 100 nM MitoTracker Green, fixed with 3% paraformaldehyde, permeabilized with 0.02% Triton X, and blocked with 5% BSA, followed by treatment with primary rabbit polyclonal cytochrome C antibody for 2 h at room temperature and followed by staining of Cy2-conjugated goat anti-rabbit secondary antibody for another 1 h. Cellular images obtained from monomer and aggregate were merged and observed under the fluorescence microscope (Nikon, Tokyo, Japan).

### Electron microscopy

Cell ultrastructure was observed using transmission electron microscopy. The cultured cells were fixed with 2.5% glutaraldehyde overnight at 4°C and then post-fixed in 1% OsO_4_ at room temperature for 60 min. Cells were stained with 1% uranyl acetate, dehydrated through graded acetone solution, and embedded in epon. Areas containing cells were block mounted, sectioned into 70-nm sizes and examined with an electron microscope (H-7500, Hitachi, Ibaraki, Japan).

### Malondialdehyde (MDA) assay

MDA levels were measured by using a lipid peroxidation MDA assay kit (Beyotime Biotech Nantong, China) according to the manufacturer’s protocol. Total protein content was determined through Bradford assay.

### Animals

Animal experiments were complied with Wenzhou Medical University’s Policy on the Care and Use of Laboratory Animals. All the experimental protocols were approved by the Wenzhou Medical University Animal Policy and Welfare Committee (Approved documents: wydw2014-0059). Five-week-old athymic BALB/c nu/nu female mice (18–22 g) were purchased from Vital River Laboratories (Beijing, China). Animals were housed at a constant room temperature with a 12:12 h light/dark cycle and fed with standard rodent diet and water.

### *In vivo* xenograft model

QBC-939 cells were harvested and injected subcutaneously into the right flank (1 × 10^7^ cells in 100 mL of PBS). After the tumors reached a volume of 70 ~ 140 mm^3^, the mice were treated with WZ26 (5 or 10 mg/kg) or CUR (10 mg/kg) once daily by oral administration. The tumor volumes were evaluated by measuring length (l) and width (w) and calculating the volume (V = 0.5 × l × w^2^) at the indicated time points. At the end of treatment, the animals were sacrificed under ether anesthesia, and the tumors were harvested and weighed.

### Statistical analysis

All *in vitro* experiments were performed at least triplicate. Data are expressed as mean ± SEM. All statistical analyses were performed using GraphPad Pro Prism 8.0 (GraphPad, San Diego, CA). Student’s *t*-test and two-way ANOVA were employed to analyze the statistical significance between two or more sets of data, respectively. *P* value <.05 was considered statistically significant.

## Results

### WZ26 markedly reduces the survival of cholangiocarcinoma cells

Firstly, we examined the anti-tumor effect of WZ26 on cell viability in different cholangiocarcinoma cell lines. Curcumin was used as a positive control. MTT assay showed that curcumin treatment for 24 h significantly reduced the viability of QBC-939 and HUCCA cells, in a dose-dependent manner with IC_50_ values of 8.9 μM and 10.8 μM, respectively (Figure 1b). However, RBE cells were not sensitive to curcumin treatment, with IC_50_ > 20 μM (Figure 1b). Similarly, WZ26 treatment significantly reduced cell viability of RBE, QBC-939 and HUCCA cells, with IC_50_ values of 4.7 μM, 4.8 μM, and 4.2 μM, respectively (Figure 1c). However, WZ26 at the dose of 20 μM showed no significant toxicity (IC_50_ > 20 μM) to the normal human biliary epithelial cell line, HIBEC cells (Figure 1c). All the IC_50_ values of curcumin and WZ26 were summarized in Table 1. These results indicate that WZ26 exhibited more potent toxic effects against cholangiocarcinoma cells with low toxicity in normal human biliary epithelial cells as compared to curcumin.

**Table 1.** IC_50_ values of curcumin and WZ26 against cholangiocarcinoma and normal human biliary epithelial cells.

IC_50_ (μM)	Cell Lines			
Compound	RBE	QBC-939	HUCCA	HIBEC
Curcumin	>20	8.9	10.8	-
WZ26	4.7	4.8	4.2	>20

### WZ26 significantly suppresses migration, invasion, and epithelial–mesenchymal transition of cholangiocarcinoma cells

We next examined the effect of WZ26 on migration, invasion, and epithelial–mesenchymal transition (EMT) in QBC-939 cells. Our data show that pretreatment of WZ26 markedly decreased TNF-α-induced migration of QBC-939 cells when compared to the vehicle control (Figure 1d). Furthermore, pretreatment of WZ26 reduced invasive capacity of the QBC-939 cells (Figure 1e). Also, pretreatment of WZ26 reversed the TNF-α-induced transcription of EMT markers such as CK-19, vimentin, and S100A4 in QBC-939 cells at the mRNA levels (Figure 1f). Finally, pretreatment of WZ26 significantly decreased the expression of E-cadherin in QBC-939 cells (Figure 1g). In summary, these results indicate that WZ26 prevented the TNF-α-induced migration, invasion, and EMT in CCA cancer cells.

### WZ26 induces apoptosis and cell cycle arrest in cholangiocarcinoma cells

**Figure 2.**
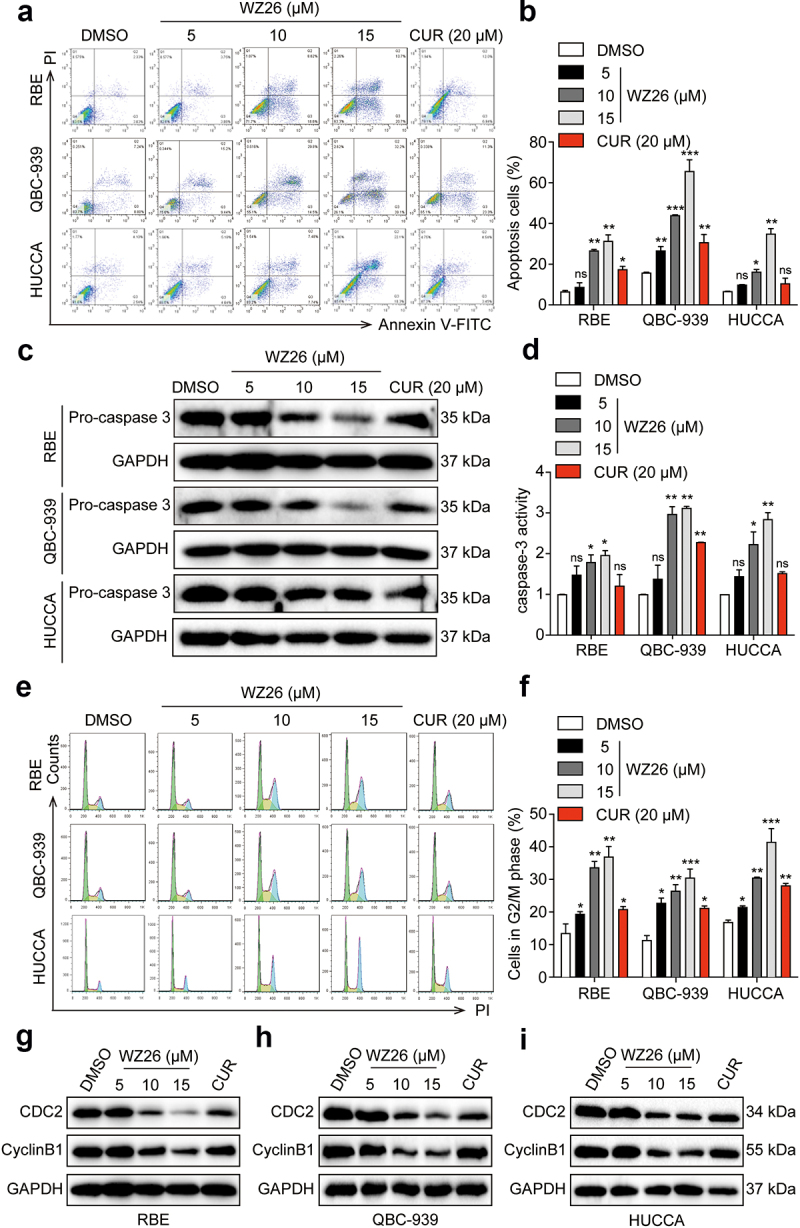
WZ26 induces apoptosis and cell cycle arrest in cholangiocarcinoma cells. Cholangiocarcinoma cells were treated with various doses of WZ26 (5, 10, or 15 µM), curcumin (CUR, 20 µM), or vehicle control (DMSO, 1 µL) for 24 h. (a) Induction of early and late apoptosis in cholangiocarcinoma cells were determined using Annexin V-FITC/PI staining. (b) The percentage of cell apoptosis was calculated and plotted. (c) Protein levels of pro-caspase 3 were determined via Western blot assay in three cholangiocarcinoma cells. GAPDH was used as the loading control. (d) Caspase-3 activity was evaluated using caspase-3 activity assay kit. (e) Cell cycle distribution was determined using flow cytometry analysis. Pictures shown were representative microphotographs of three independent experiments. (f) The percentage of cell distribution at G2/M phases was calculated. (g-i) Protein levels of CDC2 and CyclinB1 in RBE (g), QBC-939 (h), and HUCCA (i) cells were determined using Western blot and representative photos were shown. All statistical data were shown as mean ± SEM, n = 3. *, *P* < .05; **, *P* < .01; ***, *P* < .001 vs. vehicle control group.

Next, we examined whether pretreatment of WZ26 affects early and late apoptotic stages and apoptotic marker, caspase-3, in CCA cells. As shown in Figure 2a-2b, pretreatment of WZ26 dose-dependently increased the number of early and late apoptotic cells in the tested three CCA cell lines. As expected, pretreatment of WZ26 dose-dependently decreased pro-caspase-3 expression in RBE, QBC-939, and HUCCA cells as compared to curcumin-treated group (Figure 2c). In addition, pretreatment of WZ26 dose dependently increased caspase-3 activity in all the tested CCA cells as compared to curcumin (Figure 2d). These results indicate that WZ26 was more potent compared to that of curcumin-treated group in inducing cancer cell apoptosis.

Next, we examined whether WZ26 affects G2/M phase and cell cycle arrest-related markers including CDC2 and CyclinB1 in CCA cells. Pretreatment of WZ26 and curcumin induced G2/M phase arrested in CCA cells (Figure 2e-2f). Also, pretreatment of WZ26 dose-dependently decreased the expression of both CDC2 and CyclinB1 in RBE (Figure 2g), QBC-939 (Figure 2h) and HUCCA (Figure 2i) cells, respectively. Similar results were observed in the curcumin-treated groups. These results indicate that WZ26 increased G2/M arrest and decreased cell cycle markers (CDC2, and CyclinB1) thereby inducing cell cycle arrest in CCA cancer cells.

### WZ26 inhibits QBC-939 cell growth through induction of ROS

**Figure 3.**
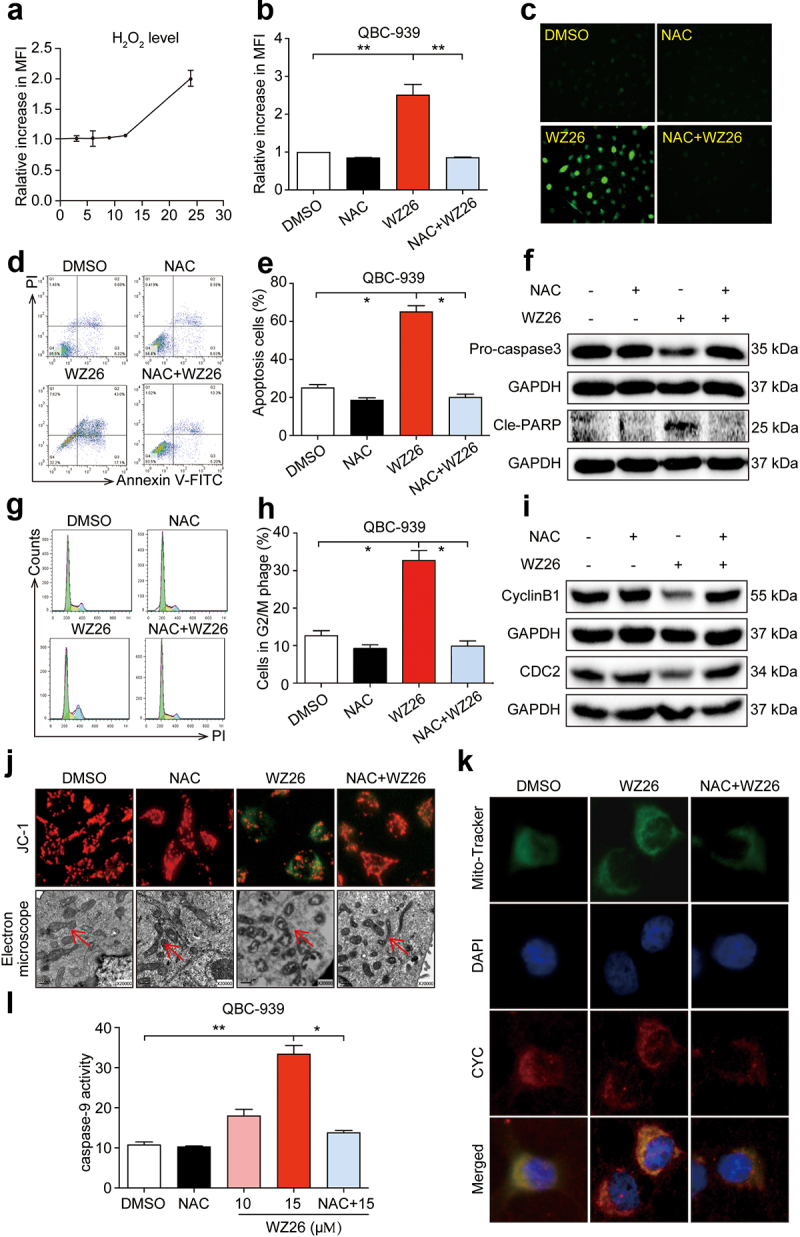
WZ26 induces apoptosis and cell cycle arrest via increasing ROS in cholangiocarcinoma cells. (a) QBC-939 cells were treated with WZ26 at dose of 15 μM for 24 h. Hydrogen peroxide (H_2_O_2_) levels in cells were detected at various time points (0, 3, 6, 9, 12, 24 h) using ROS assay kit. ROS levels were expressed as mean fluorescence intensity (MFI) in arbitrary units (AU). (b) QBC-939 cells were treated with WZ26 (15 μM) for 24 h with or without pre-treatment of NAC (10 mM, 1 h). The levels of H_2_O_2_ in cells was determined using ROS assay kit. (c) Represent images for H_2_O_2_ staining using ROS assay kit. (d-e) QBC-939 cells were treated as panel b. Apoptosis was detected using flow cytometry analysis. Representative photos (d) and statistical data (e) were shown. (f) QBC-939 cells were treated as panel b. Protein levels of pro-caspase 3 and Cle-PARP were determined using Western blot. GAPDH was used as loading control. (g-h) QBC-939 cells were treated as panel b. Cell cycle distribution was detected using flow cytometry analysis. Representative photos (g) and statistical data (h) are shown. (i) QBC-939 cells were treated as panel B. Protein levels of CyclinB1 and CDC2 were determined using Western blot. GAPDH was used as loading control. (j-l) WZ26 activates ROS-dependent mitochondrial apoptosis pathway in QBC-939 cells. QBC-939 cells were treated with WZ26 (15 μM) for 24 h with or without pre-treatment of NAC (10 mM, 1 h). (j) Representative images for Δψm detection determined by JC-1 fluorescent dyes staining as described in methods (upper row). Representative transmission electron micrographs. Swollen mitochondria were shown using arrows (lower row). (k) Representative images for cytochrome C release to cytosol analyzed by immunofluorescent staining as described in methods. Mitochondria (MitoTracker; gree (CYC, red). (l) Caspase-9 activity was determined using caspase-9 assay kit. All statistical data were shown as mean ± SEM, n = 3. *, *P* < .05; **, *P* < .01; ***, *P* < .001.

Cancer cells are more susceptible to the drastic increase of ROS, which has been reported to be involved in cell cycle arrest and apoptosis.^14^ We next investigated whether WZ26 inhibited the cell growth through induction of ROS in QBC-939 cancer cells. As shown in Figure 3a, ROS generation induced by WZ26 pretreatment was noticed at the later time period peak at 24 h in QBC-939 cells as detected by flow cytometry. Next, a ROS scavenger, NAC, was applied to identify the role of ROS in mediating the anti-cancer effect of WZ26. As shown in Figure 3b, NAC pretreatment significantly decreased WZ26-induced intracellular ROS levels in QBC-939 cells as compared to the vehicle control. Similarly, NAC pretreatment markedly reduced WZ26-increased ROS levels as detected using DCF fluorescence staining (Figure 3c). Also, pretratment of NAC alleviated cell apoptosis (Figure 3d-3e) and cell cycle arrest (Figure 3g-3h) in QBC-939 cells that caused by WZ26 treatment. Similar to these results, we found that pretreatment of NAC significantly decreased the protein levels of cle-PARP (Figure 3f), while upregulated the expression of pro-caspase 3 (Figure 3f), CyclinB1, and CDC2 (Figure 3i). These results indicate that WZ26 significantly induced ROS levels in QBC-939 cancer cells, thereby causing apoptosis and cell cycle arrest in CCA cancer cells.

### WZ26 activates ROS-dependent mitochondrial apoptosis pathway in QBC-939 cells

Increased oxidative stress-associated loss of mitochondrial transmembrane potential has been reported to be involved in the initiation of cell death.^25,26^ We next investigated the effects of WZ26 on ROS-associated mitochondrial apoptosis with or without NAC in QBC-939 cells. We evaluated mitochondrial transmembrane potential (Δψm) changes in QBC-939 cells using potential sensitive dyes JC-1. As shown in Figure 3j, WZ26 pretreatment resulted in a shift in JC-1 fluorescence from red to green as a monomeric JC-1 in the cytoplasm that collapsed Δψm. However, NAC pretreatment significantly protected against the collapse of Δψm as observed using fluorescence microscopy (Figure 3j, supplementary Fig. S1).

Next, we examined the morphology changes of mitochondria in QBC-939 cells using a transmission electron microscope. Treatment of WZ26 in QBC-939 cells induced swollen mitochondria with cristae collapsed structure and swollen vesicular matrix that represented the mitochondrial membrane destruction. NAC treatment reversed these changes (Figure 3j). In addition, we investigated the release of mitochondrial cytochrome c to cytosol using immunofluorescent staining. As shown in Figure 3k, WZ26 treatment caused cytochrome c (CYC) to diffuse from mitochondria into cytosol of the cells. Also, WZ26 treatment increased the activity of caspase-9 in QBC-939 cells (Figure 3l). All these changes were remarkably reversed by pretreatment with NAC (Figure 3k-3l). Collectively, these results indicate that redox imbalance induced by the WZ26 plays an important role in mitochondrial dysfunction and cell death in CCA cancer cells.

### WZ26 inhibits STAT3 pathway in cholangiocarcinoma cells

**Figure 4.**
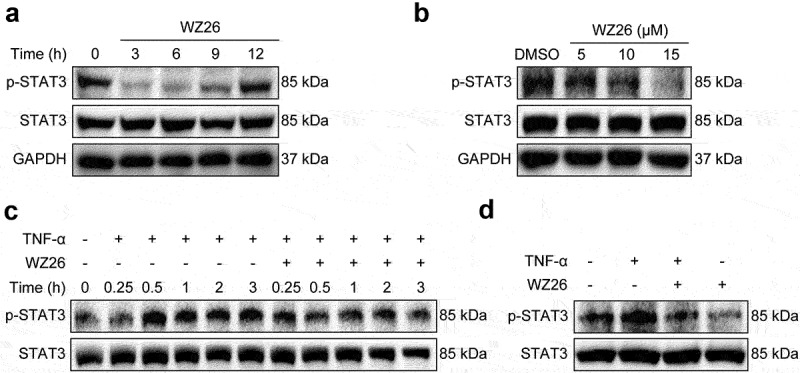
WZ26 inhibits STAT3 signaling pathway in cholangiocarcinoma cells. (a) QBC-939 cells were treated with WZ26 (15 μM) for indicated time. (b) QBC-939 cells were treated with WZ26 (5, 10, or 15 µM) for 3 h. The protein levels of p-STAT3 and STAT3 were detected using Western blot. GAPDH was used as loading control. (c) QBC-939 cells were treated with WZ26 (15 μM) for 30 min, followed by treatment of TNF-α (10 ng/mL) for indicated time. (d) QBC-939 cells were treated with WZ26 (15 μM) for 30 min, followed by treatment of TNF-α (10 ng/mL) for 3 h.

Constitutively activated STAT3 signaling regulates multiple genes related to CCA development.^27^ Thus, we investigated whether STAT3 signaling was involved in the anti-tumor effect of WZ26. As expected, we found increased levels of phosphorylated STAT3 in QBC-939 cells, indicating the activation of STAT3 signaling in CCA cells (Figure 4a). WZ26 treatment significantly reduced the levels of phosphorylated STAT3 in QBC-939 cells as comparable to the untreated cells in a dose-dependent manner within 9 h (Figure 4a-b). In addition, WZ26 treatment also markedly reduced the levels of phosphorylated STAT3 induced by TNF-α in a time-dependent manner (Figure 4c-d). These results indicate that WZ26 might exhibit anti-cancer effects via inhibiting STAT3 signaling pathway.

### WZ26 inhibits QBC-939 xenograft tumor growth *in* *vivo*

**Figure 5.**
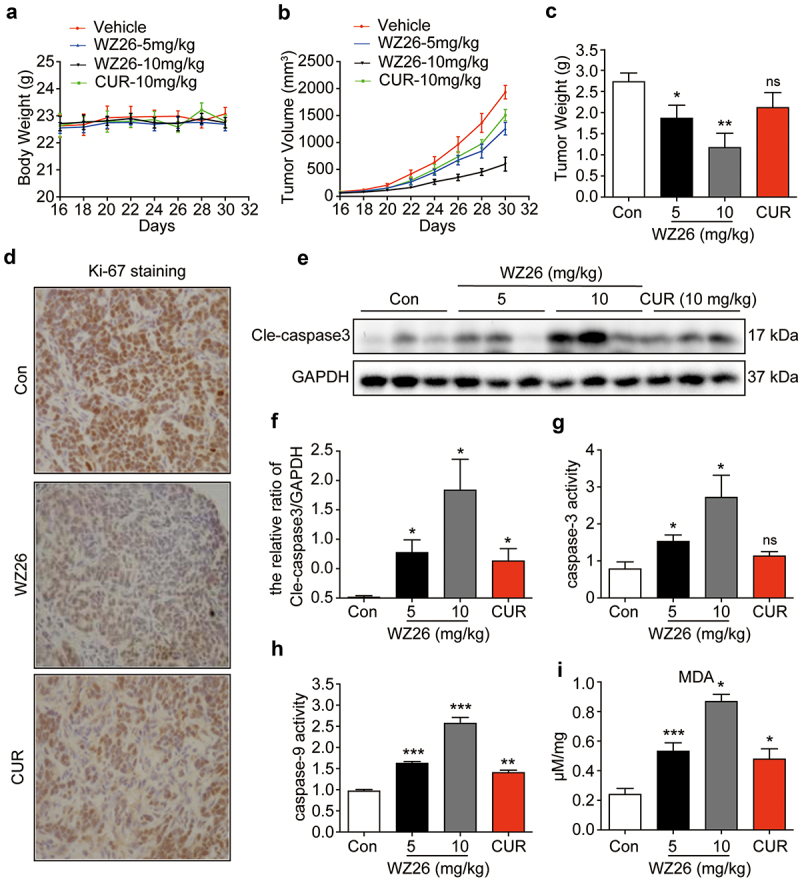
WZ26 inhibits xenograft tumor growth *in vivo*. Xenograft tumor mouse model was developed using QBC-939 cells. Body weight (a) and tumor volume (b) were quantified at various time points. (c) Mice were sacrificed on day 30, and the tumors were collected and measured. (d) The expression of Ki67 (brown dots) in xenograft tumors was determined using immunohistochemical staining. Representative images from three independent experiments were shown. (e) Protein levels of Cle-caspase 3 in xenograft tumors was evaluated using Western blot. The statistical data were shown (f). (g-h) The activities of caspase-3 and -9 in xenograft tumors were evaluated using caspase-3 activity assay kit. (i) The concentration of ROS in xenograft tumors was evaluated using lipid peroxidation MDA assay kit. All the statistical data were shown as mean ± SEM, n = 5. *, *P* < .05; **, *P* < .01; ***, *P* < .001 vs. vehicle control group.

Next, we further confirmed our *in vitro* findings in a CCA xenograft mouse model. QBC-939 cells were injected subcutaneously into the right flank of BALB/c nude mice. Once the tumor reached the volume of 70–140 mm^3^, mice were orally treated with WZ26 (5 or 10 mg/kg), curcumin (10 mg/kg), or vehicle control (CMC-Na, 100 µL) once daily for 14 days. As shown in Figure 5a, there is no significant decrease in body weight after WZ26 or curcumin treatment when compared to the vehicle control, indicating that no significant toxicity was observed of these compounds. However, WZ26 treatment markedly reduced the tumor volume (Figure 5b, supplementary Fig. S2) and tumor weight (Figure 5c). The anti-tumor effects of WZ26, at the dose of 10 mg/kg, were even better than that of the curcumin-treated group at the same dose (Figure 5c, supplementary Fig. S2). In addition, immunohistochemical staining showed that WZ26 treatment reduced the number of Ki-67 positive cells in xenograft tumors (brown dots) as compared to vehicle and curcumin-treated groups (Figure 5d). These results indicate that WZ26 inhibited the proliferation of CCA cancer cells *in vivo*. Furthermore, western blotting analysis revealed increased cle-caspase-3 expression in WZ26-treated group as compared to vehicle control (Figure 5e-5f). As expected, treatment of WZ26 (5 mg/kg and 10 mg/kg) both doses significantly increased the activities of caspase-3 and -9 in xenograft tumors (Figure 5g-5h). Similarly, treatment of WZ26 increased the concentration of ROS marker, MDA, in xenograft tumors (Figure 5i). All these effects of WZ26 were much stronger when compared to those of curcumin-treated group at the same dose (Figure 5d-i). Taken together, these results indicate that WZ26 could inhibit QBC-939 xenograft tumor growth *in vivo* via inducing ROS.

## Discussion

Despite recent advances in treatment option for the highly heterogeneous CCA, there is no significant improvement in cancer prevention has been successful over the years.^1,2^ In recent years, increasing attention has been paid to structurally modify analog in preclinical studies against CCA.^28,29^ A large number of literatures have demonstrated that curcumin can inhibit the growth of tumor *in vivo* and *in vitro*, via inducing apoptosis in various tumor cells. However, the low bioavailability of curcumin prevents its use in chemotherapeutic applications. Thus, many researches focus on improving the bioavailability of curcumin via designing and synthesizing curcumin analogs. In this context, we previously reported curcumin analogs exerted anti-cancer potential by targeting cell signaling.^30,31^ In the present study, we found that a novel curcumin analog, WZ26, exhibited improved anti-proliferative effects than that of curcumin at the same concentration, both *in vitro* (Figure 1b-1c) and *in vivo* (Figure 5b-5d), indicating the inhibitory effect of WZ26 in the CCA cell growth. In addition, WZ26 treatment dramatically suppressed cell migration, cell invasion, and the expression of E-cadherin, all of which correlated with the decrease in EMT (Figure 1d-1g). Then, we observed that WZ26 significantly induced cell apoptosis and cell cycle arrest in the tested three CCA cancer cell lines, accompanied with the corresponding changes in the levels of proteins involved in cell apoptosis- and cycle-related cascades (Figure 2), suggesting that the anti-proliferation effect of WZ26 might be due to the induction of cell apoptosis and cell cycle arrest in CCA cells. We noticed that WZ26 significantly decreased STAT3 and dreadfully induced ROS levels, which regulated mitochondrial apoptosis and inhibited cancer cell growth in CCA cancer cells. Collectively, our findings provide evidence that WZ26 is a potent and safe cancer preventive agent for CCA cancer chemotherapy.

It is known that ROS at physiological conditions functions as a second messenger and regulates intracellular signaling. A moderate increase in ROS in the cancer cells can promote cell proliferation, migration, and invasion.^7^ However, extensive scientific data report that perturbation of the redox system in the cell, balanced by the increase of ROS, leads to oxidative stress.^14,16^ Consequently, the maintenance of redox homeostasis plays a vital role in cell growth and survival.^32^ On the one hand, ROS generated at basal levels stimulate tumor development milieu in cholangiocytes.^6^ On the other hand, the dreadful increase of ROS, which is induced by the xenobiotics, increases oxidative stress in turn causing oxidative damage and promoting apoptosis and cell cycle arrest.^14,32^ In our study, we found that WZ26 increased peroxides (H_2_O_2_) in a time-dependent manner (Figure 3a-3b). Similarly, increased ROS levels were observed in WZ26 treated with DCF fluorescence stained cells (Figure 3c). Recent reports have indicated that oxidative stress affects the cell cycle by affecting the expression of cyclins.^14,33^ The control of cell-cycle progression in cancer cells is one of the effective therapeutic strategies to halt tumor growth.^34^ Our results indicate that WZ26 is an effective therapeutic strategy in the cell cycle arrest via blocking the G2/M phase and CDC2/CyclinB1 protein levels. In line with our data, WZ26 treated cancer cells increased the accumulation of G2/M phase and CDC2/CyclinB1 protein levels in a ROS-dependent manner, while treatment with NAC significantly normalized these changes. At apoptosis inducing concentrations (15 µM), WZ26 induces cell apoptosis and cell cycle arrest (Figure 3d-3i). Furthermore, blockage of ROS by NAC totally abolished the above changes induced by WZ26 in QBC-939 cells. These findings indicated that WZ26 increases ROS greater than basal levels that could induce apoptosis and cell cycle arrest in the CCA cells.

Increased oxidative stress can cause mitochondrial membrane potential (Δψm) loss, leading to the destruction of their fringe, which in turn causes CYC release.^25,26^ Recent report indicates that the depolarization of ΔΨm leads to mitochondrial swelling, uncoupling of oxidative phosphorylation, ATP depletion, and resulting in cell death.^35^ Consistent with the above findings, we observed Δψm loss and swollen mitochondria and release of CYC in the WZ26-treated QBC-939 cells, while treatment of NAC reversed these changes (Figure 3j-3k). It has been reported that several different types of genes regulated the mitochondrial-apoptosis in response to various stimuli.^36^ The released CYC subsequently activates caspase-9 that triggers effector caspase-3 and ultimately drives apoptosis.^37–39^ Much evidences indicated that many chemotherapeutic agents exerted apoptotic action via induction of ROS in CCA.^26,38,40^ In line with the above data, we found that WZ26 treatment increased caspase-3 activity in QBC-939-cells and xenograft tumors (Figure 5e-5g). Similarly, WZ26 treatment increased the activity of caspase-9 in xenograft tumors (Figure 5h). Moreover, WZ26 possessed increased apoptotic activity as compared to curcumin. These findings indicated that the curcumin analog, WZ26, shows stronger anti-tumor effects as compared to curcumin at the same dose.

In addition, WZ26 treated cancer cells increased the accumulation of G2/M phase and CDC2/CyclinB1 protein levels in a ROS-dependent manner (Figure 2g-2h). Recent reports have indicated that oxidative stress affects the cell cycle by affecting the expression of cyclins.^14,33^ Controlling cell-cycle progression in cancer cells is one of the effective therapeutic strategies to halt tumor growth.^34^ In line with our data, WZ26 is an effective therapeutic strategy in the cell cycle arrest via blocking the G2/M phase and CDC2/CyclinB1 protein levels (Figure 3i). However, further experiments are needed to reveal the exact molecular mechanism between ROS and cell cycle progression.

STAT3 plays a functional significance in initiating a pro-carcinogenic microenvironment in response to inflammation and induces oncogenesis malignancy.^41,42^ A recent report suggested that STAT3 expression and inflammation both remain as cornerstone factors of the survival and growth in cholangiocytes.^9^ Aberrant and persistent phosphorylation of STAT3 has been observed in human epithelial in intrahepatic CCA patients.^43^ Moreover, based on preclinical studies, targeting STAT3 is a highly promising approach in the treatment of CCA.^44^ Extensive scientific reports indicate that inhibition of STAT3 activation reduces the downstream genes including vimentin and other proteins involved in the migration, invasion, and EMT.^45,46^ Activation of STAT3 signaling induces cell proliferation through binding to CDC2 and stimulates CyclinB1 and other regulatory proteins.^12,47^ Also, p-STAT3 downregulation regulates the epithelial cell apoptosis in human CCA cancer.^11^ A recent report indicated that curcumin analog, which targets the STAT3 domain, exerted potent and selective antitumor activity in breast cancer.^48^ Indeed, our results reveal that WZ26 treatment inhibited phosphorylation STAT3 in a time and dose-dependent in QBC-939 CCA cells. Our findings provide evidence that WZ26 may target the STAT3 thereby inducing apoptosis and cell cycle arrest (Figure 4). A limitation of our study is that further research is needed to reveal the underlying the mechanism how WZ26 targets STAT3 in CCA cells.

Last, we have found that WZ26 also exhibited anti-inflammatory effects in LPS-stimulated macrophages. As shown in supplementary Fig. S3, WZ26 dose-dependently reduced the expression of TNF-α and IL-6 in LPS-challenged RAW264.7 cells. In addition, WZ26 reduced the protein levels of p-STAT3 that was induced by LPS. Since tumor-associated macrophages exhibit their protumor functions via inducing inflammation, the anti-inflammatory effects of WZ26 in RAW264.7 might help to synergy the anti-tumor effects of WZ26 in CCA cancer microenvironment (supplementary Fig. S4). However, further experiments are needed to confirm the immunosuppression effect of WZ26 in CCA *in vivo* model.

In conclusion, our findings demonstrate that WZ26, as a curcumin optimized analog, can inhibit the migration, invasion, and EMT of cholangiocarcinoma cells induced by TNF-α. Besides, WZ26 significantly increased ROS in the CCA cancer cells, thereby inducing mitochondrial apoptosis and inhibiting cancer cell growth both *in vitro* and *in vivo*. We further showed WZ26 effectively inhibited the activation of STAT3 in CCA cancer cells. Our results collectively suggest that WZ26 induced apoptotic cell death and cell cycle arrest in CCA cancer cells via both inhibition of STAT3 activation and induction of ROS. In addition, we also show that WZ26 exhibited good anti-tumor effects *in vivo*. In summary, our findings provide evidence that curcumin analog WZ26 could be a potential candidate against CCA and may provide a new approach to the CCA therapy.

## Supplementary Material

Supplemental Material

## Data Availability

All data used or analyzed during the current study are within the article and its supplementary materials.

## Abbreviations

AU, arbitrary units; CCA, cholangiocarcinoma; CMC-Na, carboxymethylcellulose sodium-Na; CUR, curcumin; EMT, epithelial–mesenchymal transition; MFI, mean fluorescence intensity; MMP, mitochondrial membrane potential; MTT, 3-(4,5-di-methylthiazol-2-yl)-2,5-diphenyl-2 tetrazolium bromide; NAC, N-acetyl cysteine; ROS, reactive oxygen species; STAT3, signal transducer and activator of transcription 3; TrxR1, thioredoxin reductase 1; Δψm, mitochondrial membrane potential.
