# Supplementary material for: Curcumin analog WZ26 induces ROS and cell death via inhibition of STAT3 in cholangiocarcinoma
Source: Cancer Biol Ther. 2023 Jan 16;24(1):2162807. doi: 10.1080/15384047.2022.2162807 (PMC9851268; doi:10.1080/15384047.2022.2162807)
Supplement: Supplemental Material [file KCBT_A_2162807_SM2673.zip › Supplementary File_Table S1.docx]

**Curcumin analog WZ26 induces ROS and cell death via inhibition of STAT3 in cholangiocarcinoma**

**Supplementary data**

**Supplementary Table S1.** Primer sequences used for RT-qPCR analysis

| **Gene** | **Species** | **Sequence** |
| --- | --- | --- |
| CK-19 | Human | 5’- -3’ GGTCAGTGTGGAGGTGGATT |
|  |  | 5’- -3’ TCAGTAACTCGGACCTGCT |
| Vimentin | Human | 5’- -3’ GAGAACTTTGCCGTTGAAGC |
|  |  | 5’- -3’ TCCAGCAGCTTCCTGTAGGT |
| S100A4 | Human | 5’- -3’ CAAGTACTCGGGCAAAGAGG |
|  |  | 5’- -3’ GCTGTCCAAGTTGCTCATCA |
| β-actin | Human | 5’- -3’ CCTGGCACCCAGCACAAT  5’- -3’ GCCGATCCACACGGAGTACT |
