# Supplementary figures and images for: Curcumin analog WZ26 induces ROS and cell death via inhibition of STAT3 in cholangiocarcinoma
Source: Cancer Biol Ther. 2023 Jan 16;24(1):2162807. doi: 10.1080/15384047.2022.2162807 (PMC9851268; doi:10.1080/15384047.2022.2162807)

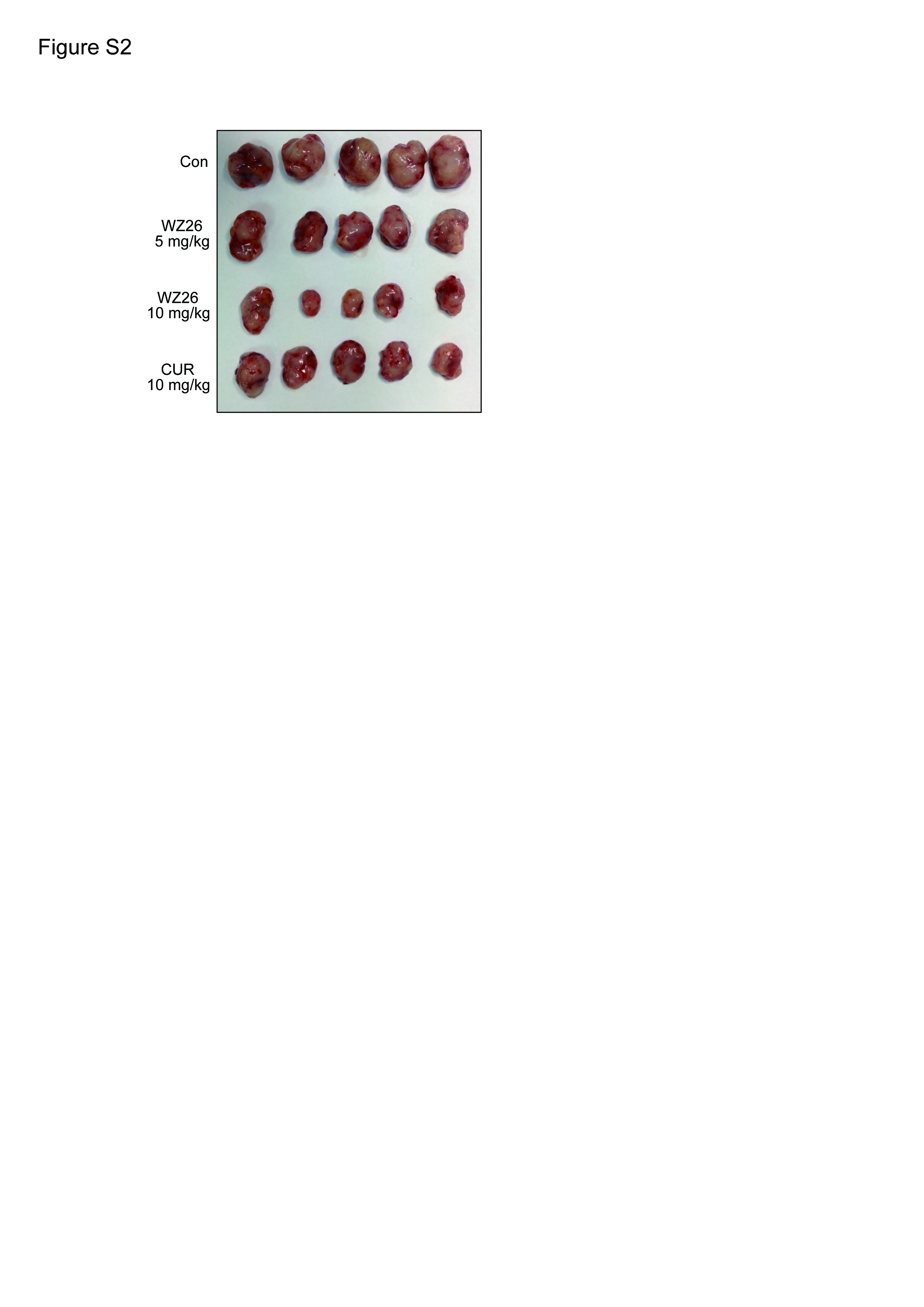

Supplement: Supplemental Material [file KCBT_A_2162807_SM2673.zip › Figure S2 20221220.tif]

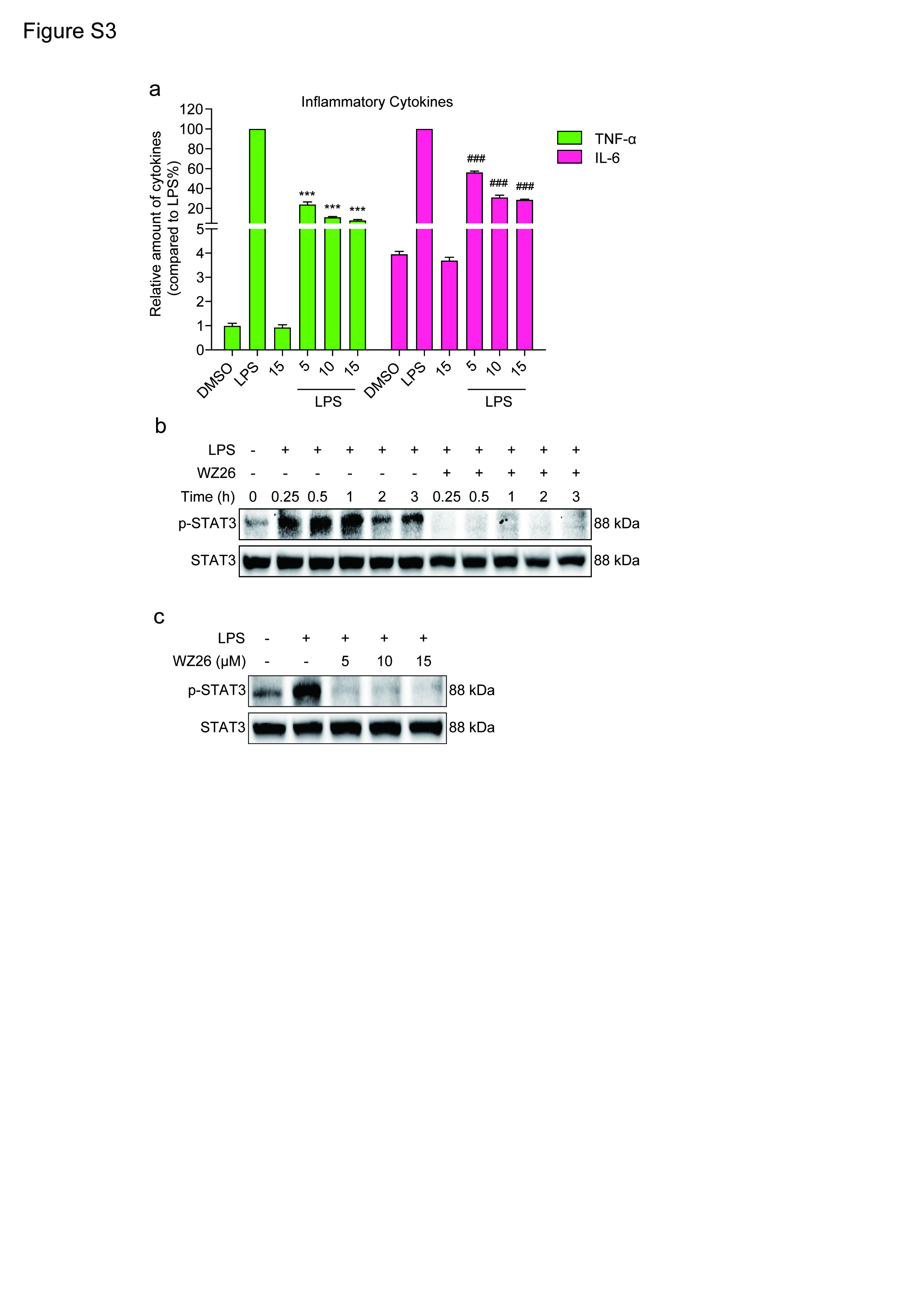

Supplement: Supplemental Material [file KCBT_A_2162807_SM2673.zip › Figure S3 20221220.tif]

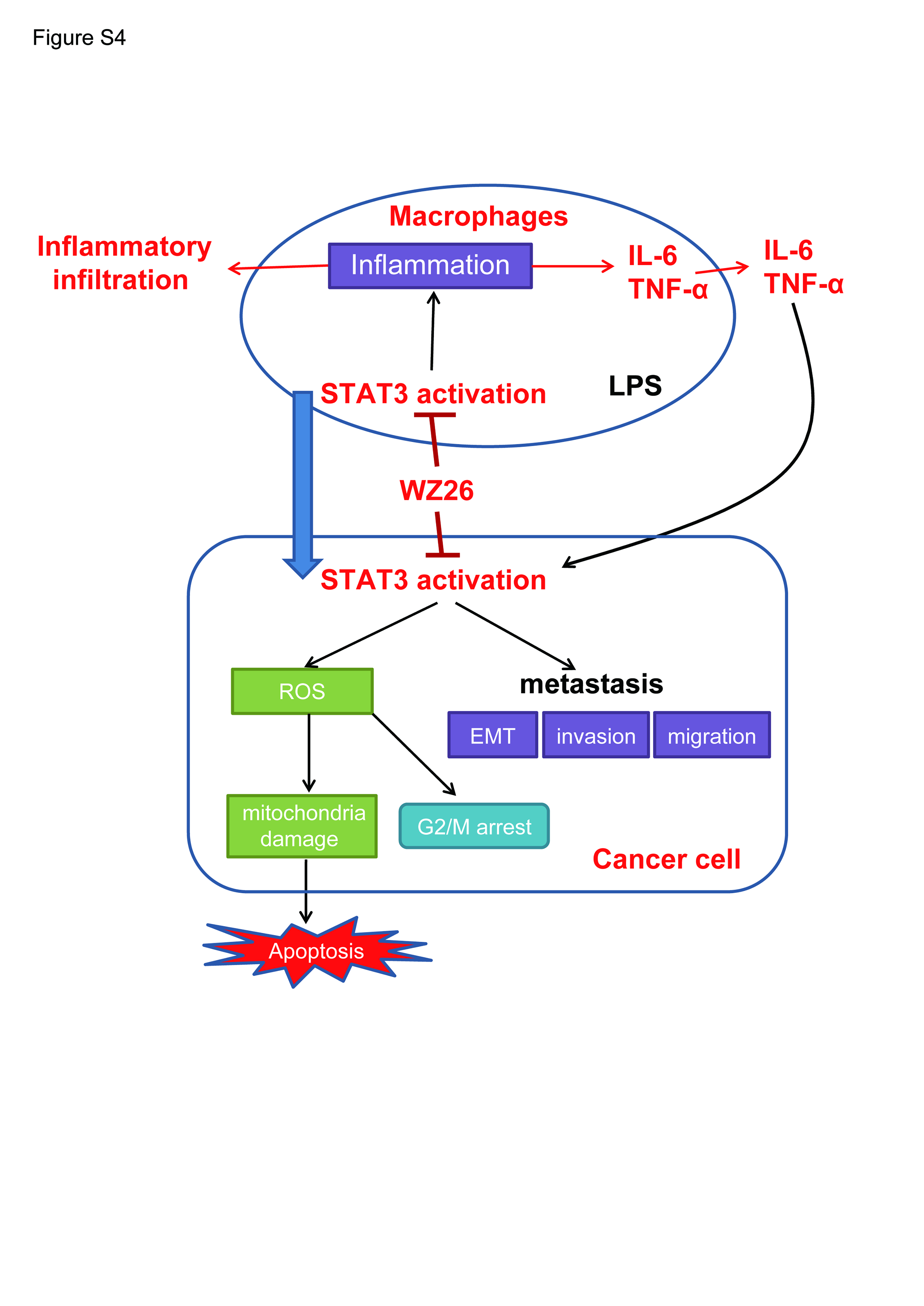

Supplement: Supplemental Material [file KCBT_A_2162807_SM2673.zip › Figure S4 20221220.tif]
